# Formative Evaluation for a Healthy Corner Store Initiative in Pitt County, North Carolina: Engaging Stakeholders for a Healthy Corner Store Initiative, Part 2

**DOI:** 10.5888/pcd10.120319

**Published:** 2013-07-18

**Authors:** Stephanie B. Jilcott Pitts, Karamie R. Bringolf, Cameron L. Lloyd, Jared T. McGuirt, Katherine K. Lawton, Jo Morgan

**Affiliations:** Author Affiliations: Karamie R. Bringolf, Cameron L. Lloyd, Jared T. McGuirt, Katherine K. Lawton, East Carolina University, Department of Public Health, Greenville, North Carolina; Jo Morgan, Pitt County Health Department, Greenville, North Carolina.

## Abstract

**Introduction:**

We examined the feasibility of increasing access to healthful food in corner stores to inform a Communities Putting Prevention to Work (CPPW) initiative by engaging stakeholders (corner store owners and customers) in a formative evaluation.

**Methods:**

Qualitative interviews were conducted with corner store owners and managers (n = 11). Customer intercept surveys (n = 179) were also conducted with customers of 9 stores. Corner stores were located in rural food deserts (municipalities without a chain supermarket) and in low-income, urban municipalities in eastern North Carolina. Interviews were transcribed verbatim and double-coded. Qualitative themes related to feasibility of increasing access to healthful foods were extracted. Shopping patterns of rural and urban customers were compared by using *t* tests.

**Results:**

Corner store owners were willing to stock more healthful foods, but they perceived that customer demand for these foods was low. Rural customers reported more frequently shopping at corner stores than urban customers and more frequently stated that the reason they do not eat more fruits and vegetables is that the stores in which they shop do not sell them. Most customers reported they would be very or somewhat likely to purchase fresh produce at a corner store.

**Conclusion:**

Corner stores may be an important source of food for rural and low-income residents and thus a good place in which to intervene. The results of this formative evaluation were used to plan and evaluate a CPPW healthy corner store initiative.

## Introduction

Obesity rates in the rural, southern United States are higher than in other US regions (1,2). The food environment may be a partial cause of high obesity rates (3). Thus, there is a need to implement environmental and policy-level supports to accessing healthful food in the rural southern United States. Because rural residents often reside farther than urban and suburban residents from supermarkets, which offer a wide selection of competitively priced healthful foods (4–8), they may rely more on corner stores for grocery shopping. For this reason, increasing access to healthful foods in corner stores may be an effective strategy to increase their consumption (9).

Results of several healthy corner store projects have suggested that increasing access to and promoting healthful items in corner stores increases sales of those items (10–13). However, to ensure that resources are used most efficiently to promote eating healthful foods and as the first step in the Centers for Disease Control and Prevention’s (CDC’s) evaluation framework (14), formative evaluation is needed to determine rural customers’ willingness to purchase healthful foods and store owners’ willingness to stock and promote more healthful foods.

In this article we describe a formative evaluation, the purpose of which was to engage stakeholders to examine the feasibility of increasing access to healthful foods in rural corner stores and to obtain baseline data for outcome evaluation. We used a mixed-methods approach by conducting qualitative interviews with corner store owners and managers to determine their perspectives on supplying and promoting more healthful foods in stores and by conducting intercept surveys with corner store customers to examine demographics, baseline fruit and vegetable consumption, shopping patterns, and purchasing habits.

## Methods

### Pitt County Communities Putting Prevention to Work partnership

This study was conducted in Pitt County (estimated population of 159,057), eastern North Carolina, which has a small urban center (estimated population of 84,986) as its county seat and is surrounded by rural agricultural areas. The Pitt County Health Department (PCHD) received a Communities Putting Prevention to Work (CPPW) grant to adopt and implement obesity prevention efforts via environmental and policy changes (15). The formative evaluation described here and in Part 1 of this series (16) was conducted to inform development and implementation of the Pitt County CPPW healthy corner store initiative. For the purposes of this article, a corner store included both convenience stores and food marts. Convenience stores were those establishments primarily engaged in the retail sale of a medium variety of canned goods, dairy products, prepackaged meats, and other grocery items in limited amounts, and food marts were stores that were similar to convenience stores in size and variety of items sold, but they were also associated with a gas station. The study was reviewed and approved by the East Carolina University Medical Center institutional review board.

### Owner and manager in-depth interviews

#### Study setting and participants

Participants were owners or managers of purposively sampled corner stores in rural food desert and urban nonfood desert municipalities in Pitt County. We defined a rural food desert as a municipality without a chain supermarket and defined urban nonfood desert municipalities as those with a chain supermarket in close proximity to neighborhoods designated as low-income by the 2000 census (17). Eligible participants were aged 18 or older, current food store owners or managers, and willing to take part in the 60-minute qualitative interview.

During February and March 2011, face-to-face qualitative, in-depth interviews were conducted with 11 food store owners and managers. Before the interview, the interviewer reviewed the informed consent document with participants and answered questions, and then participants signed the informed consent. Of the 11 stores with owners or managers interviewed, 5 were located in rural municipalities and 6 were located in the city limits of Greenville in close proximity to low-income areas. 

Interview guide questions were chosen from those published on the healthy corner stores website (18) and the New Orleans Corner Store Survey (19). The guide included questions about types of customers, acceptance of benefits from government food assistance programs (Special Supplemental Nutrition Program for Women, Infants, and Children [WIC] and Supplemental Nutrition Assistance Program [SNAP]), store products and inventory, and availability of healthful food items. The qualitative interview guide used with store owners and managers included a definition of healthful foods as “water, whole-wheat bread, low-fat milk, baked chips, fruits, and vegetables.” The final portion of the interview guide assessed the owner’s perception of the store as a part of the community and whether the food store owner would be interested in working in partnership with CPPW staff to increase access to healthful food options in the store.

#### Data management and analysis

All interviews were audio recorded and transcribed verbatim. Qualitative data were managed using NVivo version 9 (QSR International, Doncaster, Victoria, Australia). A codebook with 16 codes and operational definitions was created on the basis of interview guide questions (deductive codes) and preliminary review of 3 data-rich transcripts (inductive codes). Two coders independently coded the qualitative interview transcripts, 3 to 5 interviews at a time. Coders then met to discuss coding decisions, make revisions to the codebook, and resolve coding discrepancies. Themes related to the feasibility of increasing access to healthful foods in corner stores were determined on the basis of the frequency of similar responses.

### Customer intercept surveys

#### Study setting and participants

During March and April 2011, customer intercept surveys were conducted at 9 of the 11 stores where the owner qualitative interviews were conducted. Upon completion of the interviews, university partners asked permission to survey customers. Surveys were not conducted at 2 of the 11 stores because of low customer volume in 1 store and because corporate headquarters needed to approve customer surveys in the other. A total of 179 surveys were conducted (20 surveys per store in 8 stores [n = 160] and 19 surveys in the ninth store). To be eligible to participate, customers had to be at least 18 years of age and a self-described regular customer of the store. Informed consent for this portion of the study was waived, because no identifying information was collected from customers.

The survey contained 33 questions and took approximately 10 minutes to complete. Customers were approached and asked if they were willing to participate. If willing, they completed the intercept survey, which included questions to assess customer demographics, food stores used by customers, reasons for using these stores, and how often customers shop at corner stores. Questions also assessed how customers traveled to the store, food items they would like to be sold at the store, and what would prompt them to buy more groceries at the corner store. The survey also assessed fruit and vegetable consumption, types of food items customers currently purchase, and willingness to buy fruits and vegetables (fresh and canned) at corner stores. The customer intercept survey asked questions about purchase and consumption of the following foods: fruits, vegetables, low-fat milk, diet soda, 100% juice, baked chips, and whole-wheat bread. Customers were classified as “rural” if shopping at a rural, food desert corner store and “urban” if shopping at an urban, nonfood desert corner store. There were no rural nonfood desert stores, and there were no urban food desert stores. There were 80 customers surveyed in 4 urban stores, and 99 surveyed in 5 rural stores.

#### Data management and analysis

The survey instrument was designed using Teleform software (version 10.7, Hewlett Packard, Palo Alto, California), and completed surveys were scanned and verified; responses were electronically populated into a database. Simple descriptive statistics (means and frequencies) were calculated, and χ^2^ tests (for categorical variables) and *t* tests (for continuous variables) were used to examine differences in responses between rural and urban corner store customers. All analyses were conducted using SAS version 9.2 (SAS Institute, Cary, North Carolina).

## Results

### Owner/manager in-depth interviews

Emergent themes included 1) customer types, 2) customer preferences, 3) SNAP/WIC availability, 4) healthful items, and 5) community partnerships (Table 1). Quotes are verbatim and include dialect.

**Table 1:** Themes and Supporting Quotes From Qualitative Interviews With 11 Corner Store Owners and Managers,^a^ North Carolina, 2011

Theme	Quotes to Support Theme
Customer types	“Working class, family class, students, mothers.” (participant no. 08 [P08], urban)
	“That’s 95% college students, if not more.” (P09, urban)

Customer preferences	“What I’m saying is my clientele is not looking for something low fat. When they come into a grill they are looking for something with some grease in it.” (P06, rural)
	“I had tomatoes, it didn’t do really good. The only things they ask for is onions and stuff. Because they would rather go to the grocery center.” (P07, rural)
	“I personally do not get a lot of people asking for fresh fruit.” (P09, urban)

Food items sold most frequently	“The ones that sells . . . ones that keep. Like . . . pork and beans, franks, Vienna sausages . . . that stuff for snacks that moves pretty rapidly but . . . canned foods, they move but . . . not like what I call snack stuff.” (P02, rural)
	“The grill. That’s what keeps our store going is the grill.” (P06, rural)

Food items sold least frequently	“Grocery items, because they would rather go to the supermarkets, it’s cheaper for them, you know.” (P03, rural)
	“Well I sell a little bit of all of it. It’s kinda a slow time of the year now. It’s been slow this winter with the economy.” (P10, urban)

SNAP/WIC availability	“Well, really I won’t planning on staying here this long . . . I won’t planning on it. But if I stay here, I will get the food stamps.” (P02, rural)
	“I mean sometimes we will have a customer ask for something, and we don’t carry it, I will get it. . . . Like the WIC, pretty much everybody ask me.” (P04, urban)
	“I’m very old fashioned; I do not deal with computers at all.” (P06, rural)
	“Not yet, we are in the process of getting it.” (P11, urban)

Healthful items	“Well you see, to have fresh produce you have to have equipment for it. As you can see, the store is already crowded as it is. And I don’t have that much people asking for it…” (P03, rural)
	“Yea, I think there’s some baked chips . . . we don’t sell that much whole wheat bread. People around here like the grease and the fat.” (P05, rural)
	“Certain people buy them. It depends on what people are looking for. Most are looking for fried food.” (P07, rural)

Abbreviations: SNAP, Supplemental Nutrition Assistance Program; WIC, Special Supplemental Nutrition Program for Women, Infants, and Children.

a Quotes included are verbatim and include dialect.

#### Customer types: “We’ve got a good mix of all of ’em really”

When participants were asked about frequent customers, many said they had a good mix of school children, retirees, and employed people. One store manager said, “In the morning time we have our regular old people who come in and do their coffee thing. Then we have our regular people that come in at 7:00 because it’s time for their beer. I mean the kids going to school . . . they come in before school to get their breakfast . . . so it just depends on what time of day it is. . . . Right now it’s mostly workers who are on break for lunch” (participant no. 05 [P05], rural).

#### Customer preferences: “Snacks: chips, little cakes, candy”

When participants were asked what items they sold the most, almost half responded that alcohol and cigarettes were most popular. When further prompted with what food items they sold the most, participants reported that snack foods were most popular. Location also influenced the type and amount of items sold. One store manager responded, “Well . . . I have a school up here, so I sell a lot of drinks, soft drinks and candy, gum. . . . Now when they get off work I see a lot of beer and cigarettes” (P03, rural).

Owners and managers stated that snack foods were sold more quickly than canned grocery items. Owners and managers stated that grocery items were sold infrequently; they perceived that most customers would rather purchase grocery items more cheaply at other food venues. None of the store owners or managers interviewed said that healthful items were among the most popular items.

#### SNAP/WIC availability: “There wasn’t enough call for it”

Acceptance of SNAP and WIC benefits may be important to provide financial assistance to people living in low-income areas, where all stores were located. However, 8 of the 11 stores did not accept SNAP or WIC. When asked why they did not accept SNAP or WIC, 2 participants responded, “There wasn’t enough call for it” (P01, rural; P03, rural). Two participants said they were interested in accepting SNAP and WIC but have not filed the application because of “laziness” (P05, rural; P07, rural). Additional barriers to accepting SNAP and WIC were noted, including minimum inventory requirements: “You have to have a lot of stuff in the store like baby food and cereal . . . and most people go to the grocery store for that” (P10, urban). Of the stores that did accept SNAP and WIC, one store owner said it was because “[they] are asking for it” (P08, urban).

#### Healthy items: “This is really not a place to come if you’re looking to eat super healthy”

Every store stocked some healthful items such as fruits, vegetables, and whole-grain bread. Results were mixed when store owners and managers were asked how the healthful items were selling. One store owner said that “in the summer time [healthful items do] pretty good. [They] don’t seem to do as well in the winter” (P09, urban). In contrast, another interview participant stated, “It depends on the time of year. Everybody has gardens out here. If people have gardens, you ain’t selling no produce. So . . . you throw away a lot” (P06, rural). For the stores in which the more healthful items were not selling as well, a store manager commented, “Everything else is just really slow. Nobody likes to eat that. They don’t like to be slim . . . but some [customers] do ask for them” (P04, urban).

Overall, customer preference played a role in the healthful foods that were made available in the store. One store manager, when asked if customers request healthful items, responded, “Usually . . . people know this is a convenient store. You know, they don’t look for stuff like that. And also . . . room-wise, you know, you need a cooler to carry those items. And we don’t have the space for it” (P04, urban).

### Community partnership

Of the 11 store owners interviewed, 10 were interested in working with the CPPW community partnership to provide more healthful food options in stores. (The 1 store manager who was hesitant about partnerships needed to obtain corporate approval.) One store manager suggested, “Yeah, I think the thing to do would be to start small. . . . We need to see if we can make a dollar and not throw away 50. I mean I don’t even know if people know what a salad is around here” (P04, urban). Others said they would like to work together to get more suggestions of things to try in the stores. One manager said, “[M]aybe I’m doing it wrong. . . . We are in the South here, really it’s very hard . . . down here their mind is set up for fried pork chops, fried chicken” (P03, rural). Overall, store owners were interested in stocking and promoting more healthful items but noted they needed assistance.

### Customer intercept surveys

Rural corner store customers were older, more likely to be white, had fewer children, and were less likely to report receiving SNAP benefits than urban customers (Table 2). Urban customers reported more frequently than rural customers that they did not eat more fruits and vegetables because they liked to eat other foods more.

**Table 2:** Characteristics of 179 Rural Food Desert and Urban Nonfood Desert Customers in 9 Corner Stores, North Carolina, 2011

Characteristic	Total Value (n = 179)	Urban Customers (n = 80)	Rural Customers (n = 99)	*P* Value
**Mean age, y (SD)**	42.2 (15.8)	35.1 (14.1)	47.9 (14.9)	<.001
**Mean (SD) number of household members (including children)**	3.1 (14)	3.1 (1.5)	3.0 (1.4)	.84
**Mean (SD) number of children younger than 12**	0.7 (1.0)	0.8 (1.0)	0.5 (0.9)	.05
**Mean (SD) number of minutes participants live from corner store**	7.6 (9.5)	7.8 (9.8)	7.5 (9.3)	.84
**Mean (SD) servings of fruit eaten by participants in the past 24 hours**	1.9 (1.6)	1.9 (1.6)	1.8 (1.6)	.60
**Mean (SD) servings of vegetables eaten by participants in the past 24 hours**	1.7 (1.3)	1.6 (1.3)	1.8 (1.3)	.25
**Sex, %**				
Female	38.6	43.8	34.3	.20
Male	61.5	56.3	65.7	
**Race, %**				
African American	56.2	68.8	45.9	.002
White	37.6	23.8	49.0	
Other	6.2	7.5	5.1	
**WIC, %**	8.4	11.3	6.1	.21
**SNAP, %**	33.5	43.8	25.3	.009
**“I eat enough fruits and vegetables everyday,” % yes**	55.3	51.3	58.6	.33
**Reasons why participants do not eat more fruits and vegetables, % yes**				
I like to eat other foods more	21.2	28.8	15.2	.03
Fruits and vegetables are too expensive	8.9	10.0	8.1	.66
I don’t know how to prepare them	1.7	2.5	1.0	.33
I don’t have time to prepare them	10.6	13.8	8.1	.22
The stores where I do most of my shopping don’t sell them	3.9	2.5	5.1	.22
They are of poor quality at the stores where I do most of my shopping	1.7	1.3	2.0	.41
Other	11.7	11.3	12.1	.85

Abbreviation: SD, standard deviation; WIC, Special Supplemental Nutrition Program for Women, Infants, and Children; SNAP, Supplemental Nutrition Assistance Program.

Most customers shopped at grocery, corner, and dollar stores (Table 3). Rural customers reported shopping at corner stores more frequently than urban customers. Urban customers were more likely to walk to get to the corner store than rural customers, who were more likely to drive or bicycle to the store. Most customers (92%) said they would be very or somewhat likely to purchase fresh fruit at a corner store. The most common fresh fruits identified were apples, oranges, bananas, and grapes. Most customers (65%) said they would be very or somewhat likely to consider buying canned fruit at corner stores. When asked about buying fresh vegetables at corner stores, 79% of customers said they would be very or somewhat likely to do so. The 4 most common fresh vegetables customers identified were collards, cabbage, carrots, and string or green beans. Finally, 65% of customers said they would be very or somewhat likely to consider buying canned vegetables at corner stores.

**Table 3:** Shopping Patterns of 179 Customers in 9 Corner Stores, North Carolina, 2011

Shopping Pattern	All Customers (n = 179)	Urban Customers (n = 80)	Rural Customers (n = 99)	*P* Value

	%			
**Store types where customers shop**				
Grocery store	99.4	100.0	99.0	.55
Corner store	96.1	96.3	96.0	.30
Dollar store	78.2	78.8	77.8	.88
Drug store	41.9	42.5	41.4	.88
Farmers market	35.2	31.3	38.4	.32
**Reasons for shopping at store where customers most frequently buy food**				
It has good prices	84.4	87.5	81.8	.30
It has good quality	82.1	86.3	78.8	.20
It has a good selection of items	76.0	88.8	65.7	<.001
It’s close to where you live	73.2	75.0	71.7	.62
It is clean	71.5	82.5	62.6	.003
**How often do participants shop for food at corner stores?**				
Never	0.6	1.2	0	.05
A few times a year	1.1	2.5	0	
Once a month	8.4	13.8	4.0	
Once every 2 weeks	6.7	7.5	6.1	
1 or 2 times per week	35.2	30.0	39.4	
More than 5 times per week	48.0	45.0	50.5	
**Transportation to store**				
Walk	18.4	27.5	11.1	.02
Bicycle	1.7	0	3.0	
Car (own or that of household member)	70.4	63.8	75.8	
Car (that of nonhousehold member)	8.9	8.8	9.1	
Bus	0	0	0	
Other	0.6	0	1.0	
**What would help more customers buy more groceries at corner stores**				
Needs to have a wider selection	58.7	66.3	52.5	.06
Better prices	49.7	46.3	52.5	.40
Better quality	17.9	25.0	12.1	.03
Need a more convenient way to get to the store	9.5	7.5	11.1	.41
It needs to be cleaner	5.6	10.0	2.0	.04

## Discussion

Our formative evaluation suggests that corner store owners are willing to stock more healthful foods but that they perceive customer demand of these foods to be low, indicating the need for price promotions to help create demand for more healthful options. Price promotions were encouraged as a part of the Pitt County CPPW healthy corner store initiative. We also found that store owners had not completed the paperwork to become SNAP or WIC certified due to self-described “laziness,” although many corner store customers surveyed were SNAP or WIC beneficiaries. Therefore, the Pitt County CPPW healthy corner store liaison has provided technical assistance to owners to become SNAP and WIC certified.

Regarding selling fresh produce, owners expressed concern about space, equipment, short shelf-life, and low customer demand, but most expressed willingness to try to stock more healthful items. Our qualitative findings are similar to those of Song et al (11), who found that if corner store owners stock and sell healthful items regularly, they are more likely to continue stocking those items because they are responsive to consumer demand. Our work and that of others indicate that lower supply of healthful foods in corner stores is a reflection of actual or perceived customer preferences for less healthful alternatives (20), as opposed to store owners’ lack of willingness to stock more healthful foods.

Compared with urban customers, rural customers more frequently reported they did not eat more fruits and vegetables because the stores where they do most of their shopping do not sell them. Rural customers reported shopping at corner stores more frequently than urban customers. Taken together, these findings support the notion that rural corner stores are a critical element of the food environment in which to intervene to provide more healthful food items.

The formative data described here and in Part 1 of this report aided the Pitt County CPPW team in identifying which store to use to pilot the healthy corner store initiative. We provided the CPPW team with a list of the 11 corner stores ranked in order from most to least likely to be a successful partner. The corner store ranked first — due to the owner’s willingness to stock more healthful foods, the store’s proximity to low-income housing, and the store’s large SNAP and WIC customer volume — became the CPPW pilot healthy corner store. We also compiled and hand-delivered corner store reports to the 11 stores, with a letter thanking them for participation, to build rapport with store owners.

Customer intercept survey data indicated that customers decide in which store to shop on the basis of price, selection, and quality. Many customers stated that they would buy more groceries at the corner store if it had a wider selection. Therefore, in the pilot store, the owner stocked a variety of produce items, beyond the traditional apples, bananas, and oranges.

Limitations of this study include the use of purposive and convenience sampling and the small sample of owners or managers and customers. The use of convenience sampling potentially introduced volunteer and response biases. Interviewers may have been more likely to approach customers if they looked as if they were not in a rush, possibly introducing bias. Customers may have felt compelled to answer favorably about eating healthfully because of the nature of the survey. However, we did get unfavorable responses related to healthful items, suggesting that participants were responding truthfully. Strengths of this study include the potential for creating community partnerships and the use of formative evaluation data to plan and implement future CPPW healthy corner store initiatives, increasing the likelihood of long-term success and sustainability.

Further evaluation is needed to examine the business operations of corner stores to determine whether varying the mix of foods is beneficial to both the store customers and owners. Others (9,13) have found that increasing access to and promotion of healthful food items using shelf labels, taste tests, and food demonstrations have increased sales of more healthful foods in corner stores. The work described in this article informed a CPPW (15) healthy corner store initiative, which included a local program goal of increasing access to more healthful foods throughout rural and underserved areas, with participating stores agreeing to do the following: 1) increase the number and type of healthful options available, 2) rearrange produce placement to increase visibility of the product, 3) lower the price of healthful options, and 4) participate in in-store promotion of healthful options. Ultimately, evaluation of the Pitt County CPPW healthy corner store initiative should guide future work to increase access to healthful foods in rural and underserved areas.
